# High Dependency Units (HDUs) in Pediatrics: Need of the Hour in Resource-Limited Settings

**DOI:** 10.7759/cureus.67755

**Published:** 2024-08-25

**Authors:** Sidhant Pundhir, Milind R Shinde, Srikanta Basu

**Affiliations:** 1 Pediatrics, Lady Hardinge Medical College, New Delhi, IND

**Keywords:** resource limited setting, critical care, pediatrics, hdu care, picu care

## Abstract

Background

Critically ill children, being vulnerable and having higher mortality as compared to adults, require specialized intensive care. However, the focus of critical care remains on adults, especially in resource-limited countries. Limited beds in the pediatric intensive care unit (PICU) along with the limitation of infrastructure and staff add to the challenge in pediatric critical care. In such scenarios, high-dependency units (HDUs) can help save a few more lives, who could not be provided with the PICU facility. HDU provides a level of care that is intermediate to that of the PICU and the general ward providing close observation, monitoring, and intervention to children who are critically ill. Our study highlighted that critically ill children can be given a chance of survival in resource-limited settings through HDU care.

Materials and methods

In our single-center prospective observational study, 204 children (less than 18 years) admitted to the HDU over 11 months and fulfilling the inclusion criteria were included. Blood samples were drawn for baseline investigations. The child's clinical course in the HDU along with the total duration of stay were recorded in a proforma. Children were reviewed for the requirement of invasive, non-invasive respiratory support along with inotropic support. Various parameters of the pediatric risk of mortality (PRISM) IV score were recorded within a time period of two hours prior and four hours following admission to HDU. The final outcome of the children was recorded. All data were analyzed and reviewed.

Results

Among the 204 patients admitted to HDU 136 (66.7%) children were treated successfully, whereas 63 (30.9%) children succumbed to their disease and complications, and five children were transferred to the PICU. Among various factors of age less than one year, the primary indication of admission being respiratory distress, the need of >2 inotropes had higher odds of mortality. Odds of mortality were eight times in patients with shock and altered sensorium, three times in children with respiratory distress, and two times in those having seizures. Those patients with a PRISM IV score of >15 had almost 100 times higher odds of mortality as compared to those with a score of <15.

Conclusion

In a resource-limited setting like ours, there’s a scarcity of PICU beds for the provision of critical care. We envisage that providing intensive care in HDU will help save a few more lives, who could not be provided PICU facility for any reason.

## Introduction

Pediatric critical care has been showing promising growth in developing and low-middle-income countries. The development of pediatric intensive care units (PICU) with continuous monitoring and intervention by trained intensivists and residents has improved outcomes in critically ill children. Despite tremendous growth in the development of PICUs, challenges are faced in terms of infrastructure development, capital investment, training of healthcare workers, and cost of treatment. In government-run institutions, the availability of an adequately equipped PICU is limited as compared to the patient load these institutions cater to [1,2]. In our country, the provision of pediatric intensive care is poor or non-existent in rural India and overcrowding of PICUs in urban settings is common. In such a scenario, the establishment of a high-dependency unit (HDU) can reduce the burden on the limited number of beds available in the PICU of tertiary centers where sick patients are referred [3,4]. The gap between the number of beds available in PICUs and number of patients requiring critical can be overcome in developing and low-middle income countries by the establishment of an HDU. The HDU provides a level of care that is intermediate to that of the PICU and general ward, providing close observation, monitoring, and intervention to children who are, or have a significant potential to be, physiologically unstable. As compared to the PICU (1:1), HDU has a relaxed nurse-to-patient ratio (1:3). In our setup, which is a hospital for children only, there is an HDU with a ventilation facility (both invasive and non-invasive), which sets it apart from many other centers with no such provision of mechanical ventilation [5]

Since the concept of HDU is a relatively new practice in pediatric practice, especially in resource-limited settings, we planned this study to observe the patient profile and outcome in our HDU over one year and to look at the alternative model for caring for sick children in resource-limited settings.

## Materials and methods

A single-center, prospective observational study was conducted in the department of pediatrics at Kalawati Saran Children's Hospital affiliated with Lady Hardinge Medical College located in New Delhi, India. Approval from the Institutional Ethics Committee (LHMC/IESC/2019/16) was obtained before the study. Children who were admitted to the HDU at our tertiary care center were included in the study with informed consent. Children who had a stay of less than 24 hours in the HDU and were admitted for a procedure or post-procedural observation were excluded from our study. Also, children who received an invasive form of ventilation prior to referral to our center with an endotracheal tube in situ or who received cardiopulmonary resuscitation elsewhere and were referred to our center were excluded from our study. A total of 204 children less than 18 years old admitted to the HDU were enrolled in the study from October 2019 to September 2020.

The sociodemographic profile, anthropometric parameters, and immunization status were recorded as per the proforma. Vital parameters including heart rate, respiratory rate, blood pressure, oxygen saturation, and temperature were recorded at admission and during the hospital course. Various parameters of the Pediatric Risk of Mortality (PRISM) IV score, which included clinical, biochemical, hematological, and physiological parameters were recorded within two hours prior and four hours following admission to the HDU. Blood samples were drawn for baseline investigations (serum electrolytes, urea, creatinine, complete blood count, and coagulation profile).

The child's clinical course in HDU along with the total duration of stay was recorded. After admission to the HDU, the methods of oxygen supplementation and the need for ventilatory support along with its duration were recorded. For children who required inotropic support, records were maintained regarding the number, duration, and dose of inotropes required during the stay.

Data was also recorded regarding the development of sepsis, systemic inflammatory response syndrome (SIRS), and multiorgan dysfunction syndrome (MODS). A note was made about the requirement and duration of antibiotics.

The final outcome was analyzed as death, discharge from the hospital, left against medical advice, or transfer from the hospital.

All relevant data were recorded in the predesigned proforma. All data were compiled in a Microsoft Excel spreadsheet (Microsoft Corporation, Redmond, WA, US) and analyzed using SSPS software v.23 (IBM Corp., Armonk, NY, US). Sensitivity, specificity, positive predictive value, negative predictive value, and odds ratio were the validation tests used in this study. A p-value of <0.001 was considered significant. Outcome and predictive score characteristics were determined with 95% confidence intervals(CI).

## Results

In our study, among the children admitted to the HDU, 136/204 (66.7%) children survived, whereas 63/204(30.9 %) succumbed to their disease and complications. Five (2.4%) children were transferred to the PICU. Children under five years of age showed the highest mortality among age groups and were statistically significant with a p-value of <0.001 (Table 1). Male children had a higher proportion among admissions but the impact of sex on outcome was not statistically significant. Unvaccinated children showed higher mortality and children who were vaccinated fully or partially showed a higher survival rate, which was statistically significant (p<0.001). Poor outcomes were observed in patients with stunting, which was statistically significant (p<0.001).

**Table 1:** Demographic variables of study participants (N=204) MUAC: mid-upper arm circumference

VARIABLES		NON-SURVIVORS		SURVIVORS		CHI-SQUARE TEST P-VALUE
		NO	%	NO	%	
Age	<1 month	9	14.3%	0	0.0%	<0.001
	1 month - 1 year	21	33.3%	32	22.7%	
	1 - 5 years	17	27.0%	88	62.4%	
	>5 years	16	25.4%	21	14.9%	
Sex	Male	41	65.1%	90	63.8%	0.863
	Female	22	34.9%	51	36.2%	
Vaccination	Vaccinated	4	6.3%	25	17.7%	<0.001
	Partially Vaccinated	49	77.8%	114	80.9%	
	Not Vaccinated	10	15.9%	2	1.4%	
Weight for age	-1 TO +1 SD	1	2.9%	21	17.5%	0.002
	> -1 SD	12	34.3%	13	10.8%	
	> -2 SD	12	34.3%	37	30.8%	
	> -3 SD	10	28.6%	49	40.8%	
	> +1 SD	0	0.0%	0	0.0%	
	> +2 SD	0	0.0%	0	0.0%	
	> +3 SD	0	0.0%	0	0.0%	
Height/length for age	-1 TO +1 SD	4	11.4%	54	45.0%	<0.001
	> -3 SD	11	31.4%	15	12.5%	
	> -2 SD	19	54.3%	41	34.2%	
	> -1 SD	1	2.9%	10	8.3%	
	> +1 SD	0	0.0%	0	0.0%	
	> +2 SD	0	0.0%	0	0.0%	
	> +3 SD	0	0.0%	0	0.0%	
MUAC	<11.5 CM	9	31.0%	9	7.6%	<0.001
	11.5 - 12.5 CM	14	48.3%	49	41.2%	
	>12.5 CM	6	20.7%	61	51.3%	

Among admitted children, respiratory distress was the most common indication for admission to the HDU followed by shock, altered sensorium, and seizures (Table 2). We could provide ventilatory support in the HDU to 66 children, out of which 27/66 (40.9 %) survived. Some form of inotropic support was required in 139/204 (68.1%) of the children.

**Table 2:** Indication of admission to the HDU among survivors and non-survivors & ventilatory support provided (N=204) p-value <0.001 considered significant HDU: high dependency unit

VARIABLES		NON-SURVIVORS		SURVIVORS		CHI-SQUARE TEST P-VALUE
		No	%	No	%	
Respiratory distress	Yes	53	84.1%	88	62.4%	0.002
	No	10	15.9%	53	37.6%	
Shock	Yes	55	87.3%	67	47.5%	<0.001
	No	8	12.7%	74	52.5%	
Altered sensorium	Yes	41	65.1%	28	19.9%	<0.001
	No	22	34.9%	113	80.1%	
Seizures	Yes	26	41.3%	37	26.2%	0.032
	No	37	58.7%	104	73.8%	
Bleeding	Yes	9	14.3%	8	5.7%	0.040
	No	54	85.7%	133	94.3%	
	No	1	1.6%	111	78.7%	
Ventilatory support	Yes	39	61.9%	27	19.9%	<0.001
	No	24	38.1%	114	80.1%	

Among the complications observed, 198/204 (97.1%) of the enrolled patients had features suggestive of systemic inflammatory response syndrome (SIRS), and 154/204 (75.5%) had sepsis. Severe sepsis was present in 142/204 (69.6%) of the enrolled patients.

The PRISM IV score is a mortality prediction score, with parameters recorded two hours prior and four hours following the admission. In this study, the PRISM IV score was recorded for all enrolled patients similarly following admission to HDU. As depicted in Table 3, it was observed that almost 58/63(92%) of the non-survivors had a PRISM IV score of >15 and this difference between survivors and nonsurvivors was statistically significant, thereby indicating higher mortality with a PRISM IV score of >15.

**Table 3:** PRISMA IV scores among survivors and non-survivors (N=204) PRISM: pediatric risk of mortality score

PRISM IV	NON-SURVIVORS		SURVIVORS		P-VALUE
	No	%	No	%	
>15	58	92.1%	14	10.3%	<0.001
=15	5	7.9%	122	89.7%	
Total	63	100 %	136	100%	

**Figure 1:**
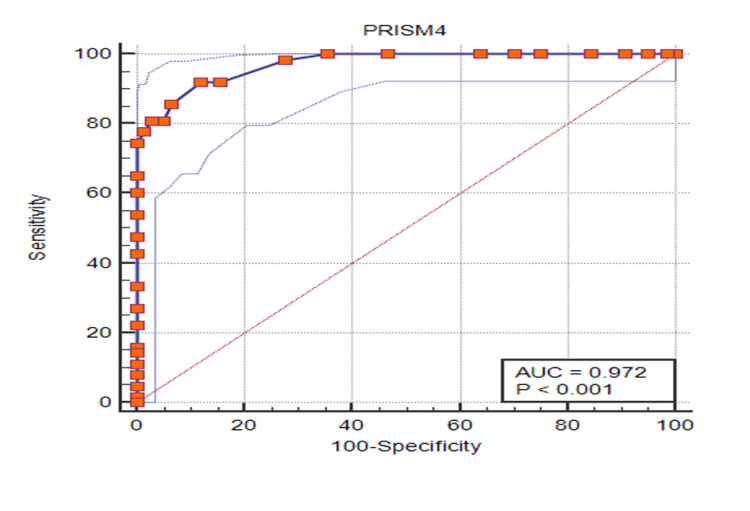
Area under the curve (AUC) for the PRISM IV score among enrolled patients (N=204) PRISM: pediatric risk of mortality

Also, for a score of 15, as depicted in Figure 1, the area under the receiver operating characteristic (ROC) curve (AUC) was 0.976 (95% CI 0.944-0.993, p=<0.0001). With the AUC for the PRISM IV score of 15 being close to 1 and a narrow confidence interval of 0.944-0.993, the PRISM IV score demonstrated good prediction for mortality as observed in this study.

With a positive likelihood ratio of 8.94, patients with a PRISM IV score of >15 are almost nine times more likely to have mortality as an outcome, as compared to those with a score of <15. With positive and negative predictive values being 80.56% and 96.06%, those with a PRISM IV score of <15 are more likely to survive.

In our study, the odds of mortality among patients with shock and altered sensorium were eight times while those with respiratory distress had three times the odds of mortality, and those with seizures had two times the odds of mortality. Those patients with a PRISM IV score of >15 had almost 100 times higher odds of mortality as compared to those with a score of <15.

## Discussion

A total of 204 patients were enrolled in the study over a period of one year. The primary outcome of the study was to evaluate the outcome of patients admitted to the HDU. The majority of the published studies analyzing the outcomes of patients were conducted in PICUs. In the absence of standard guidelines for establishing HDUs, there is a dearth of adequate studies that describe the profile, outcomes, and clinical parameters of patients admitted to HDUs. In resource-limited settings, since the number of beds in the PICU is limited, we wanted to evaluate the outcome of patients in our HDU, which has the facility of three ventilators. In most published studies, especially from India, the outcome was assessed using the PRISM III score [6,7]. Not enough studies are available on the analysis of the performance of the PRISM IV score in critical care settings across developing nations where the clinical profile of patients is different from PICUs in Western countries where this score was developed [8,9].

Among the patients admitted to HDU, the primary clinical presentation in the maximum number of patients was respiratory distress (141/204; 69.1%) followed by shock (122/204; 59.8%). The frequency of altered sensorium (69/204; 33.8%) and seizures (63/204; 30.9%) was almost similar, with bleeding being the least. These observations were similar to the findings of the study by Mukhija G et al. [10], Khilnani P et al. showed respiratory system involvement being the most frequent indication for admission to critical care settings [11]. We could provide invasive mechanical ventilation to 66/204 (32.3%)children through HDU. Previous studies have shown wide variation in patients requiring mechanical ventilation in PICUs, for example, Mukhija G et al. (68.3%) [10], Khilnani P et al. (20.68% mechanical ventilation) [11], Sahoo B et al. (6; 8.48%) [12], Singhal D et al. (8; 15%) [13], and Zhang Z et al. (48.7%) [14]. Although HDUs in the study setting had the provision of mechanical ventilators, there was a gap between the number of ventilators available and the number of patients requiring mechanical ventilation.

Among the 204 patients admitted to the HDU, mortality was observed in 63/204 (30.9%) while 136/204 (66.7%) patients were discharged and five patients were transferred to the PICU. The proportion of nonsurvivors (30.9%) in this study was similar to the study by Jyothi AK et al. where nonsurvivors were 28% [15]. While mortality in other studies conducted in PICUs by Khilnani P et al. (6.7%) [11], Shah AA et al. (18; 11.8%) [16], Singhal D et al. (18%) [13], Mukhija G et al. (8%) [10], and Zhang Z et al. (4.7%) [14] was lower as compared to our study. During the same period as our study, our hospital PICU reported 92/504 (18.2%) mortality.

The reasons for higher mortality in our HDU setup can be attributed to the non-availability of sophisticated equipment and the smaller number of healthcare workers (doctors and nursing staff) leading to inadequate intensive monitoring required for round-the-clock care. Moreover, during the COVID-19 pandemic, only COVID-negative patients were admitted to the PICU, thereby sick patients requiring PICU admission were managed in HDU. Also, being a tertiary-level hospital, Kalawati Saran Children's Hospital catered to sick patients referred for admission for critical care, who could not be admitted to other COVID-19-dedicated tertiary-level government teaching hospitals. However, our HDU was able to cater to these sick children who are deprived of intensive care in resource-limited settings, reducing the overall mortality.

Among the primary clinical presentations, The odds of mortality among patients with shock and altered sensorium were eight times while those with respiratory distress had three times the odds of mortality, and those with seizures had two times. Similar findings were observed in the study conducted by El Nawawy et al. [17] and Gulla KM et al. [18], where the most frequent indication of admission was respiratory involvement among both survivors and non-survivors, with a higher number of mortalities observed among patients with neurological involvement.

Mechanical ventilation among enrolled patients in this study had statistically significant differences between survivors and non-survivors, with poor outcomes amongst mechanically ventilated patients. This observation of higher mortality among mechanically ventilated patients is similar to that observed in a study by Thukral et al. [19], though mortality in this study was higher (60% vs 40%). This could be ascribed to the fact that the study by Thukral et al. was conducted in the PICU with better monitoring and resources as compared to the HDU. Our study showed that providing ventilators in HDUs can improve outcomes in critically ill children and can help save a few more lives who couldn’t receive ICU care due to resource-limited settings.

In our study, it was observed that the use of vasopressor drugs and that too ≥2 vasopressor drugs had poor outcomes with a higher number of mortalities among patients who were administered the same. These observations were similar to those observed in a study by Costa et al. [20].

The majority of the patients in our study had sepsis 154/204 (75%), with mortality observed in 61/154 (40%) of sepsis-positive patients. Similar mortality (44%) was observed among patients with sepsis in a study conducted by Singhal et al. [13], while mortality among patients with sepsis varied from 31% by Sahoo et al. [12] to 6% by Zhang et al. [14]. This higher mortality among sepsis patients, as compared to the PICU, could be ascribed to better asepsis maintained in PICUs.

The PRISM IV score is a recently developed mortality prediction score and is yet to be widely incorporated in critical care settings of developing countries [21,22]. In our study, the AUC for PRISM IV (Score =15) was a 0.976 confidence interval of 0.944-0.993. The study conducted by Zhang et al. [13] had AUC 0.76. This was a multicentric study conducted across PICUs located in China comparing the performance of PRISM IV. In our study, almost 58/63 (92%) of the patients who could not survive had a score of >15, with the odds of mortality being almost 100 times as compared to those with a score of < 15.

Even in the regression analysis, a PRISM IV score > 15 showed an independent correlation with mortality as an outcome. With high sensitivity and specificity of around 90%, a positive likelihood ratio of around 8, and a negative likelihood ratio of 0.9, the PRISM IV score can be used in patients for predicting mortality. In the studies by Costa et al. [20] and Pieracci FM et al. [23], the risk factors for death among patients in the PICU were MODS (multiorgan dysfunction syndrome) on admission, use of mechanical ventilation, use of vasoactive drugs, number of vasoactive drugs, presence of nosocomial infections, and duration of PICU stay, which was also observed in our study.

Current evidence shows that critical care in resource-limited nations, although growing, still focuses mainly on adults [24]. Our study highlighted that children being more vulnerable than adults, with high mortality rates, can be given a chance to survive in resource-limited settings through HDU care.

As it is an observational study from a particular HDU, the limitations of our study include that we could not compare survival outcomes with those of PICU patients. Also, a longer follow-up after discharge, around three to four months, to assess the sequelae and complications, would have been a better predictor of the quality of critical care provided in our HDU. Moreover, we did not anticipate the COVID-19 pandemic while designing our study, so some of our findings may not be reflective of the general population, as COVID-19-positive patients were admitted to dedicated wards and centers and thus could not be enrolled.

## Conclusions

In a resource-limited setting like ours, there is a scarcity of PICU beds for the provision of critical care. We envisage that the HDU can contribute significantly to intensive care and providing ventilators in HDU will help save a few more lives that could not be provided in the PICU facility for any reason in resource-limited settings. For policymakers, this study will help further strengthen the already established HDUs, especially in areas where the establishment of a PICU is not feasible.
